# Darwin's Theory of Descent with Modification, versus the Biblical Tree of Life

**DOI:** 10.1371/journal.pbio.1001096

**Published:** 2011-07-05

**Authors:** David Penny

**Affiliations:** Institute for Molecular BioSciences, Massey University, Palmerston North, New Zealand

There is a strong urban myth that Charles Darwin introduced and/or advocated a “Tree of Life” for the classification of living organisms. This has recently been highlighted by Lawton [1], but dates back to at least Doolittle [2]. It is often combined with the idea that we require a “paradigm shift” to take into account extensive lateral gene transfer, especially in prokaryotes. Yes, extensive lateral gene transfer occurs widely in prokaryotes (see [3]–[5]), and this enriches our understanding of evolution by emphasizing a more gene-centered view. In contrast, though acknowledging the prior availability of the tree of life simile, Darwin continually referred to his “theory of descent with modification”, an expression that encompasses a wide variety of fundamental processes including both vertical descent and lateral gene transfer, thus removing any need for paradigm shifts.

For Western Europe, the Tree of Life phrase is biblical in origin and, although in Darwin's words, it is “a useful simile”, it is best not interpreted literally. The basic confusion may have arisen because it is not recognised that Darwin was mainly interested in the mechanisms of evolution. These include both whether mechanisms that could be studied in the present could explain past events, and establishing the continuity between all living creatures. He was not primarily interested in the description of the patterns of evolution.

We need to reappraise the different concepts, what Darwin meant by them, and how he used the terms. The Tree of Life example is not the only case where Darwin's work has potentially been misunderstood (and I will mention some of these later). In contrast to being interested in describing patterns of relationships, I argue that Darwin appears to have been more interested in the extent to which mechanisms that can be studied in the present are both necessary, and sufficient, to explain events in the past—an approach that I interpret as being learned from his geological background (Figure 1) [6],[7].

**Figure 1:**
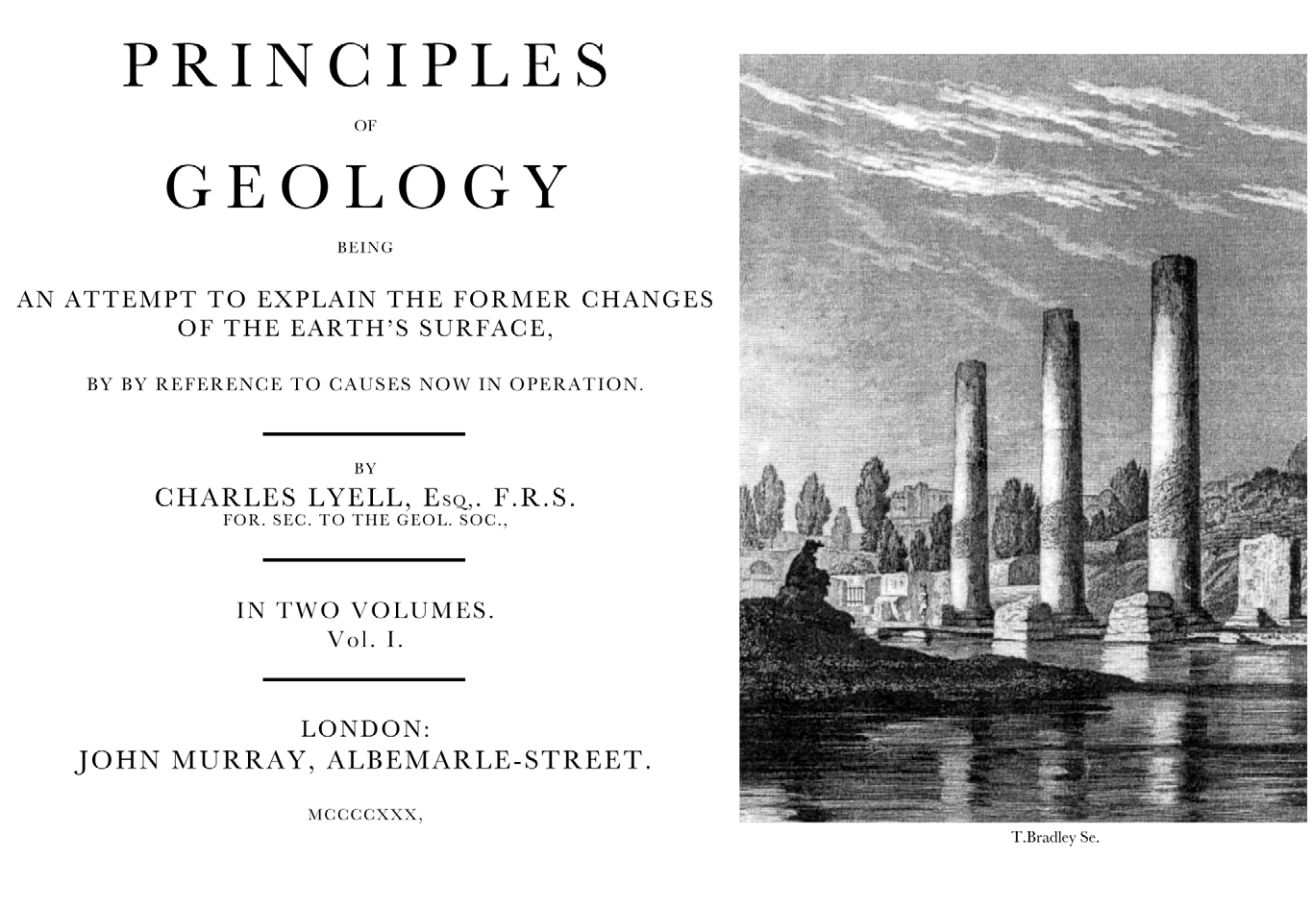
In 1830, Charles Lyell (effectively Charles Darwin's mentor) published his *Principles of Geology* as an attempt to explain past geological changes by mechanisms that can be studied in the present. Lyell's frontispiece was the remains of the ancient Greek Temple of Serapus at Puzzuoli, near Naples in Italy. Known forces of land subsidence and earthquakes could account for its unusual appearance today. It had been built on land, and then subsided over a millennium, which allowed marine molluscs to leave the high water mark. The remains of the temple had then been raised up again in an earthquake in September 1438 to its present position. It was an example of how known forces and mechanisms could be used to explain past events. Continuity was essential, but the known forces could vary in their intensity over time.

## How Old Is the Tree of Life Concept?

The Tree of Life is a biblical phrase that predates the 19th century by several millennia. In all, the phrase occurs 11 times in Genesis, Proverbs, and the Book of Revelations (http://www.biblegateway.com/quicksearch/). In Genesis, it is found three times (2∶9 and 3∶22 and 3∶24); in the middle of the Garden of Eden were both the Tree of Life and the “Tree of the knowledge of good and evil” (from which Eve supposedly ate the fruit). Eating of the fruit of the Tree of Life would apparently have given eternal life, and that eternal life theme appears again in Revelations 2∶7, and 22∶2, 14, and 19. The other references are in Proverbs (3∶18, 11∶30, 13∶12, 15∶4), where it appears more metaphorical in intent.

Thus, my first point is that Darwin certainly did not coin the phrase. Indeed, the phrase has widespread use in many cultures (see Wikipedia) and is clearly an ancient idea with strong mystical overtones. This ancient origin of the term the Tree of Life does not preclude its use in science, but it certainly means that we need to be careful how we use it—we always use it in lower case letters (tree of life) to help avoid unintended implications. It perhaps should also be pointed out that searching the same biblical Web source does not find any occurrences in either the Old or New Testament of either the “theory of descent”, nor of “descent with modification”!

## Darwin's Alternative View of “Descent with Modification”

Having established that Darwin was not the originator of the term, my second main point is that, instead of using the tree of life concept, Darwin referred to his theory as “descent with modification”. For example, in the first edition of the *Origin of Species* (Darwin 1859, see http://darwin-online.org.uk/) Darwin uses the phrases “theory of descent” and/or “descent with modification” 21 times (on pages 12, 14, 189, 206, 320, 349, 351, 354, 358, 361, 399, 429, 444, 477, 479, 484, 493, 494, 496, and 497). As such, it includes the idea of an evolutionary tree but also includes hybrids (long a standard technique for plant breeders), of which Darwin was fully aware, as well as ideas from genetics that would have been unfamiliar to him. These include mechanisms of evolution such as the transfer of genes between bacteria, or from bacteria to eukaryotes by endosymbiosis (by mitochondria and chloroplasts), and so on. No doubt there are other mechanisms, but they all fit well with the concept of descent with modification. Basically, descent with modification allows “cycles” in “graphs” (technically, a tree is a “connected graph without cycles” [8]). In real life, cycles could result from hybridisation, endosymbiotic gene transfer, lateral gene transfer, recombination, lineage sorting, the complexities of genealogical relationships, etc. Importantly, Darwin's more general phrases, including descent with modification, emphasise the continuity between populations, subspecies, sibling species, etc., which was perhaps the more fundamental issue in the mid-19th century (rather than that of the precise form of relationships).

This modification with descent phrase is used in several contexts, including main headings in the *Origin*. It occurs in the discussion around difficulties for the “theory of continuity”—including the absence or rarity of transitional varieties (leading to a discussion on the relative completeness of the fossil record, and why the fossil record should not be read literally—still excellent reading for paleontologists). It is used as an explanation for the affinities of the extinct forms of life both to each other and to living forms. Another usage is that species of different classes do not necessarily change together, or at the same rate, or to the same degree; yet, in the long run, they all undergo some modification. It is also an explanation of the naturalness of the classification of eukaryotic groups, and also an explanation for biogeographic distributions. Again, the phrase is used with reference to variability under domestication, and as an explanation for homologies. As such, descent with modification, especially in relation to continuity over short- and long-term time scales, is central to Darwin's view of evolution. All this is fully acceptable to modern evolutionists, even though it is important to acknowledge that Mendel's particulate view of inheritance was not at all well known to Darwin, and (as I note later) authors in the mid 19th century were still puzzled by the principles of inheritance.

## How Darwin Used the Concept of the Tree of Life

In contrast to the 21 occurrences of the theory of descent, in the *Origin of Species* there is only a single usage of the Tree of Life. However—and this is my third point—it is not used as a description of relationships, but rather as an analogy for competition between species (and groups of species) during evolution. It is the analogy of a branch of the tree overgrowing, and supplanting a “feebler branch” (see Darwin [9], p. 148). The full quote is “*buds give rise by growth to fresh buds, and these, if vigorous, branch out and overtop on all sides many a feebler branch, so by generation I believe it has been with the great Tree of Life, which fills with its dead and broken branches the crust of the earth, and covers the surface with its ever branching and beautiful ramifications*”. As such, it is not a description of the relationship between taxa, but rather a suggestion that a living tree analogy can be applied to lineages of species competing (and supplanting) other lineages or groups of species—whether fungi, plants, or animals. In other words, microevolutionary processes are similar in principle to macroevolutionary processes, a central part of his thinking [7]. He also comments that “*the affinities of all the beings of the same class have sometimes been represented by a great tree*”, and immediately follows with the sentence “*I believe this simile largely speaks the truth*”. And that is the usage that is best used today: the tree of life is a useful simile. But the context in the *Origin* shows that readers were already expected to know about the tree simile; it was neither a novel nor a central part of his theory.

Thus, it seems clear that Darwin deliberately chose not to use the phrase Tree of Life; he was certainly aware of it and used the phrase in his Notebooks [10] that were started over 20 years before the *Origin* was published. However, even here his usage was very different, and he suggested in his notes that the Tree of Life “*should perhaps be called the coral of life, base of branches dead; so that passages cannot be seen*”. Nevertheless, the continuity between forms of life is still there. The 21 usages of the theory of descent in the *Origin*, and the one reference to the Tree of Life (which was not as a description of relationships) demonstrates that Darwin rejected the concept of a Tree of Life to describe his views of evolution in favour of his theory of descent with modification. This does not deny that he was well aware of the concept, just that he declined to use it for his theory; instead, he focussed on mechanisms.

## A Continuous Process

This leads to a very interesting fourth main point emphasising further the concept of continuity that was mentioned earlier. Clearly, an evolutionary tree is a useful concept and Darwin was certainly aware of it, and has the well-known tree diagram in the *Origin*. However, an important function of that diagram (and one less appreciated today) was that it explicitly asserted the continuity of populations, subspecies, species, sibling species, genera, etc. At a time when separate creation of each species was the dominant theme, it was important to establish the idea that there were continuous sets of intermediates (generations and populations) between all these levels. This aspect of continuity was not unique to Darwin; it would be favoured by some versions of orthogenesis, in which evolution was thought to be driven continuously by some external force, and some evolutionary interpretations of the Great Chain of Being [11], which posits a linear hierarchy with humans (especially European males) at the top! This continuity aspect of the diagram is not now generally recognised because Darwin's continuity between species (“evolution”) was basically accepted among scientists within about a decade of the publication of the *Origin* [12]. As an aside, it is interesting that the tree is a non-binary tree, not a bifurcating tree, and this must be of concern to cladists (a small group who attempted to maintain a strict relationship between a binary tree and the formal classification).

## What Were Darwin's Intentions?

Is there any reason that Darwin basically rejected the Tree of Life phrase in favor of the theory of descent with modification? Before Darwin there were many ideas about the relationships of species [13]. Perhaps the most favoured was the Great Chain of Being [11], but there were many others that are referred to in Stevens [14] and Winsor [15], and a diagram summarising many of them is in [16].

It certainly follows that species both changed with time and could split into more than one species. Put another way, there was no “unchangeable essence” to a species—this was an “idea” from Plato that was added into the concept of species that developed in the late 17th century [17]. Given Darwin's ideas above, a tree-like form of relationship could have been a natural prediction. But, as I mentioned previously, scientists of the time already knew about hybrids, and their importance in plant breeding. Even a century earlier, hybridisation was known to Linnaeus [18]—indeed, Linnaeus suspected “genera” were the true “fixed” units, and species themselves could change and vary over time. So Darwin considered a tree a useful analogy, but he was not very interested in the tree itself. He was more interested in asserting the continuity between populations and species and genera, and on whether the mechanisms that could lead to change were sufficient. Why Darwin did not really like the tree of life concept is worth a major study in its own right, perhaps equivalent to Lovejoy's classic study on The Great Chain of Being [11]. I should emphasise again that at the time the *Origin* was published, virtually no biologist knew the basis of Mendelian genetics. Even as late as 1870 ([19] pp. 76–77), some researchers were studying whether there was any inherited (“genetic”) effect on a second foal, from a horse that fathered the first foal. Given the lack of formal genetic information at the time, descent with modification was certainly keeping options open and focussed just on what was known, and on the continuity between generations and populations.

A final aside is that, although many research groups have studied trees extensively, several research groups have long advocated an increase in the use of networks in phylogenetics. Trees (formally, “connected acyclic graphs”) are too simple to reflect all the signals in sequence data—even though a tree is generally an excellent summary of the main effects. For example, I and others have emphasised the multiple (and conflicting) signals in sequences [20], have published a program (SpectroNet) for reconstructing networks [21], and shown that assuming a strict tree leads to biases in maximum likelihood when additional signals are present [22]. This should make it clear that researchers like myself are not rejecting the tree per se but enriching the tree concept into a network. Indeed, the more mathematically focussed side of phylogenetics has long used networks (e.g., [23]) to show more complex relationships. Huson and Bryant [24] have updated the SplitsTree program, and Holland [25] discusses ways of forming consensus networks.

## Lessons from Darwin

So far I have concentrated on what was written by Darwin. My aim has been to illustrate why it is important for both evolutionary biology itself, and for our communication of the subject more widely, that Darwin's work and his concept of descent with modification is not misunderstood. It is unfortunate, however, that there are some other examples where biologists seem to have insufficient knowledge of what Darwin wrote and of his reasoning. This probably affects us all some of the time, and it is unfortunate that there is not more communication between evolutionary biological researchers, and historians of evolution who regularly study the older manuscripts [26],[27].

One example that demonstrates such a need for more communication is in regard to the neutral theory of molecular evolution, in which King and Jukes [28] suggested that neutral amino acid change was “non-Darwinian” evolution—leading to a decade of fruitless debate over naming. However, Darwin stated “*Variations neither useful nor injurious would not be affected by natural selection, and would be left either a fluctuating element, as perhaps we see in certain polymorphic species, or would ultimately become fixed*,…”. (This version is from the 6th edition of the *Origin*, and is expanded from earlier editions.) Thus, Darwin was well aware of neutral changes, “*neither useful nor injurious*”, and commented on the two aspects that eventually (when made quantitative) led to the neutral theory of molecular evolution (namely, the high level of polymorphism in natural populations, and the apparent “molecular clock” for the rate of fixation). A second example was the suggestion that Darwin favoured phyletic gradualism (over punctuated equilibria), but this claim has already been shown to be incorrect [29],[30]. He carefully differentiated between ecological and geological time scales when using terms such as “fast” or “slow”.

Another example is Mayr's interpretation of species as the fundamental unit of evolution. But, as mentioned earlier, Darwin assumed a continuum of intermediate stages between individuals, populations, varieties, subspecies, sibling species, species, subgenera, genera, and so on; there was nothing really special about the level of species per se. This was highlighted by Mallet's [31],[32] analysis that recent work with molecular data supports Darwin's interpretation. Nevertheless, many (most?) biologists still appear to consider species as a quite fundamental unit, even though Mallet (as well as other authors) have argued otherwise.

These three examples, together with that of the Tree of Life, reflect situations where some excellent evolutionary biologists were not fully aware of Darwin's thinking and reasoning. However, there is almost certainly a more general issue here in that biologists studying evolution, and historians studying the history of evolution, do not meet regularly. Most of the issues I have discussed with respect to the Tree of Life are well known to historians of biology; it is the lack of opportunity to interact between the groups that has hampered communication.

So where does this leave us? With respect to the Tree of Life, it is unambiguous that Darwin neither invented this ancient phrase, nor used it to describe the fundamentals of his evolutionary understanding. In contrast, he routinely used the term theory of descent with modification, and focussed more on the mechanisms of evolution and the continuity of life. I would therefore argue that we evolutionists need to have a better understanding of the history of our subject if we wish to claim something as novel. My own interpretation of why Darwin focused on a mechanistic view of evolution is that he started his professional career as a geologist [6],[7], effectively with Charles Lyell as his mentor [33], and Lyell sought to explain past geological events by mechanisms that could be studied in the present (Figure 1). Whether this interpretation of Lyell's influence stands future tests remains to be seen, but certainly Darwin was much more interested in mechanisms that could explain evolution than in describing patterns of relationships. As mentioned earlier, given Darwin's theory of descent and his interest in explaining the past by known mechanisms, we should welcome lateral gene transfer as another mechanism that can help explain past biology.
